# Q Fever Vertebral Osteomyelitis Complicating Vertebroplasty

**DOI:** 10.5041/RMMJ.10430

**Published:** 2021-01-19

**Authors:** Karina Dorfman, Ayelet Eran, Nesrin Ghanem-Zoubi

**Affiliations:** 1 Radiology Department, Bnai-Zion Medical Center, Haifa, Israel; 2 Department of Radiology, Rambam Health Care Campus, Haifa, Israel; 3 Infectious Diseases Institute, Rambam Health Care Campus, Haifa, Israel

**Keywords:** Q fever, *Coxiella burnetii*, paravertebral abscess, vertebral osteomyelitis

## Abstract

Query (Q) fever is a zoonotic bacterial infection caused by *Coxiella burnetii*. In a minority of patients, chronic disease can occur after acute infection. Endocarditis and infections of aneurysms or vascular prostheses are the most common forms of chronic Q fever in adults. We report a case of an elderly female patient with chronic Q fever vertebral osteomyelitis at the site of her previous cement vertebroplasty, complicated by paravertebral abscess. Patient treatment required prolonged drainage in addition to the long duration of antibiotic treatment by doxycycline and hydroxychloroquine. Osteomyelitis is a rare clinical presentation in adults with chronic Q fever. However, it is important to consider Q fever in the differential diagnosis of culture-negative osteomyelitis, especially in countries where *C. burnetii* is endemic, such as Israel.

## INTRODUCTION

Query (Q) fever is a zoonotic bacterial infection caused by *Coxiella burnetii*. The common clinical manifestations of acute Q fever include pneumonia and hepatitis. In a minority of patients (2%–5%), chronic disease can occur after symptomatic or asymptomatic acute infection. Adult patients at the highest risk for chronic Q fever are those with valvular heart disease, a vascular graft, or an arterial aneurysm. Endocarditis and infections of aneurysms or vascular prostheses are the most common forms of chronic Q fever in adults. In contrast, osteomyelitis is one of the most common findings in children with pediatric chronic Q fever.

We report a case of an elderly female patient with chronic Q fever vertebral osteomyelitis at the site of her previous cement vertebroplasty.

## CASE PRESENTATION

The patient was a 78-year-old female. Her medical background included dyslipidemia, osteoarthritis, calcium pyrophosphate deposition disease, and osteoporosis complicated by D12 compression fracture following a fall that was treated with cement vertebroplasty two years before the current presentation.

The patient presented to the emergency room with epigastric pain, anorexia, and weight loss during a period of two months, without fever. Prior endoscopic examination performed in an outpatient facility was unremarkable.

Her physical examination was notable for mild right upper quadrant tenderness without any other significant findings. Her white blood cell count was 13.5×10^3^/mL with an elevated C-reactive protein (CRP) of 72.4 mg/L; liver enzymes were within normal limits. Abdominal sonography showed mild hepatomegaly without any other pathologic findings.

**Figure 1:**
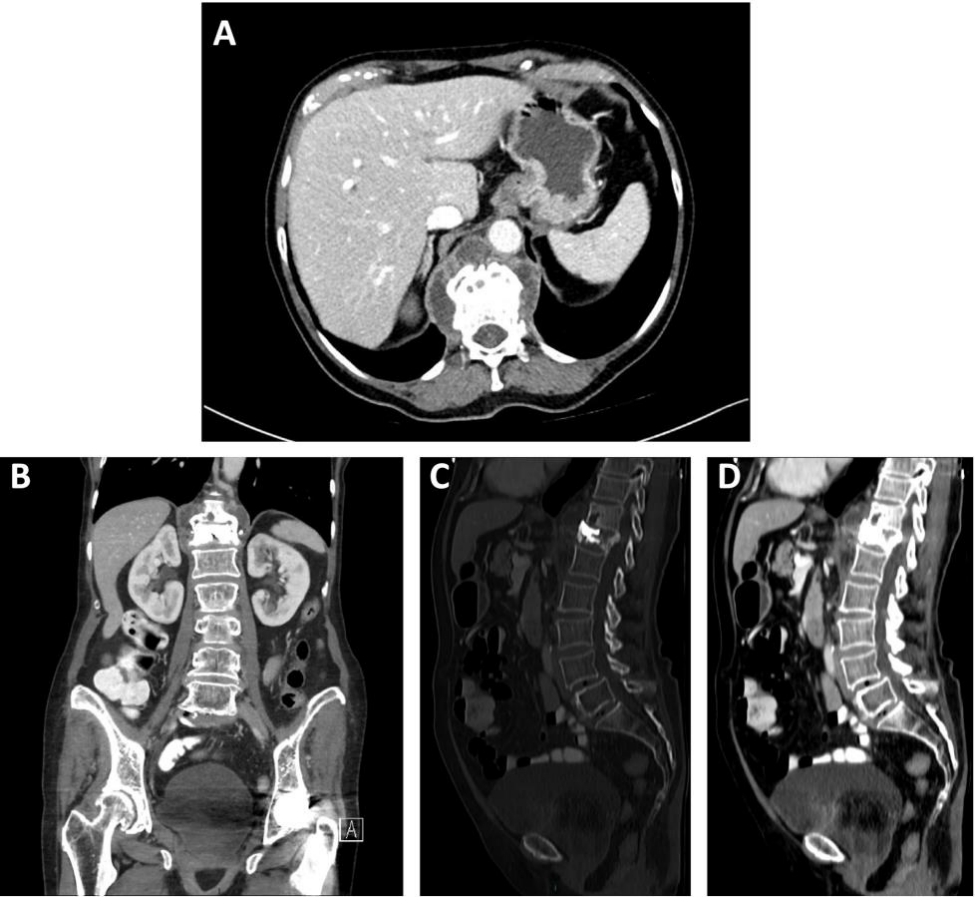
Contrast-enhanced Abdominal Images Computed Tomography Images. **A:** Axial soft tissue window at the level of D11–D12. **B:** Coronal soft tissue window. **C:** Sagittal bone window. **D:** Sagittal soft tissue window.

**Figure 2:**
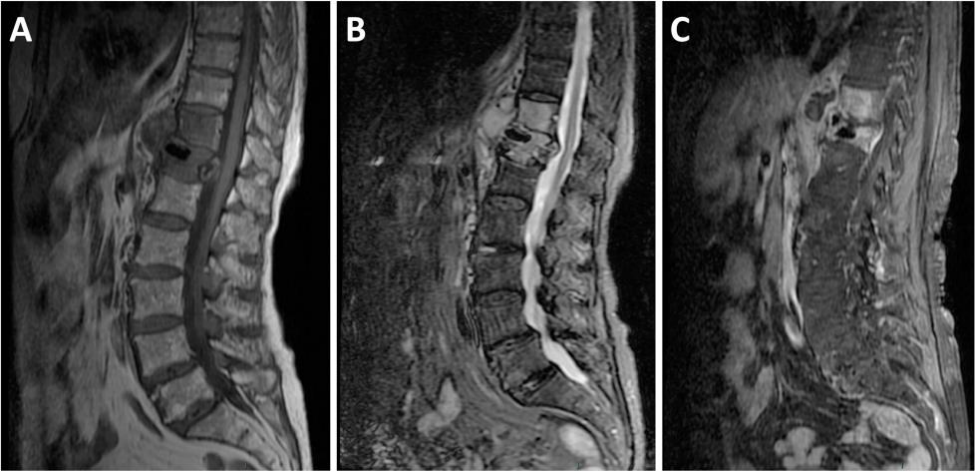
Sagittal Magnetic Resonance Imaging of the Lumbar Spine. **A:** T1-weighted non-contrast-enhanced. **B:** Short tau inversion recovery (STIR). **C:** T1-weighted fat-suppressed with Gadolinium-based contrast media.

Contrast-enhanced abdominal computed tomography (CT) revealed paravertebral collections at the D12 level—the site of her prior cement vertebroplasty (Figure 1). The D12 vertebral body was in mild collapse with cement filling. The rest of her thoracolumbar spine that was included in the abdominal CT scan (levels D9–coccyx) showed mild osteopenia and degenerative changes. Magnetic resonance imaging (MRI) examination revealed T2 and short tau inversion recovery (STIR) hyperintensity in the D11 and D12 vertebral bodies and intervertebral disc, accompanied by enhancing paravertebral collections, findings suggestive of discitis osteomyelitis (Figure 2).

Blood cultures were negative. Percutaneous CT-guided fine-needle aspiration was performed with pus aspiration, without bacterial growth from the specimen. Polymerase chain reaction (PCR) of the pus sample was positive by 16S rRNA that was sequenced to *C. burnetii* as well as with specific primers of the bacterium. Serology testing performed with indirect immunofluorescent assay (IFA) revealed high antibody titers to *C. burnetii* with phase I IgG of 3200 and phase II IgG of 1600, which is indicative of chronic Q fever. Echocardiography showed no vegetations.

The patient was started on doxycycline and hydroxychloroquine treatment, which was planned for at least 18 months. The collection was percutaneously drained continuously for 8 months and withdrawn after secretions stopped. On the follow-up MRI, the collection resolved. Clinically, at one-month follow-up, the patient was doing well.

## DISCUSSION

Common manifestations of chronic Q fever are endocarditis and vascular infection.^1^ Osteoarticular infection is a less common manifestation. It has been described in children,^2^ and as part of an extension from adjacent vascular infection in adults.^3^,^4^

Vascular and heart valve prostheses are well recognized risk factors for chronic Q fever endocarditis and vascular infection. Only a few case reports described patients with osteomyelitis at sites of prosthetic joins or surgical fixation devices. To the best of our knowledge, this is the first case report of *C. burnetii* infection at a vertebroplasty site. As primary osteoarticular infection is a rare and probably underreported presentation of chronic Q fever, the role of the previous surgical intervention is less established in these cases. Multiple studies have shown that the presence of previous cardiovascular pathology with or without surgical interventions leads to changes in the immunologic microenvironment, which creates “locus minoris resistentiae” for the *C. burnetii* infection in these sites.^5^ These mechanisms may also explain the osteoarticular infections found in sites of previous trauma or surgical intervention.

The indolent clinical course along with the imaging findings of paravertebral abscess raises a differential diagnosis that includes atypical infections like tuberculosis and brucellosis.

The present case emphasizes the importance of including Q fever in the differential diagnosis of culture-negative osteomyelitis, especially following intervention, as well as looking for etiology by molecular testing including 16S rRNA (pan-bacterial PCR). It is especially important in countries where Q fever is endemic, as is the case in Israel. According to Israel Ministry of Health weekly epidemiological reports, Q fever incidence in Israel during 2018 was approximately 2.5 per 100,000 population.^6^ Data from European Union countries for the same year showed the highest incidence of 0.7 per 100,000 population in Spain. Other European countries where the disease is endemic, like Bulgaria, France, and Germany, had an incidence between 0.1 to 0.6 per 100,000 population.^7^ These data highlight the hyper-endemic status of Q fever in Israel and the urgent need for immediate public health interventions.

Chronic Q fever infection has a slow healing course. Therefore, the recommended antimicrobial therapy of chronic Q fever infections is a very prolonged duration of at least 18 months. One of the unique features of the present case was the need for prolonged drainage for source control.

## Abbreviations

CT: computed tomography
MRI: magnetic resonance imaging
Q: query

## References

[1] Kampschreur LM, Delsing CE, Groenwold RHH (2014). Chronic Q fever in the Netherlands 5 years after the start of the Q fever epidemic: results from the Dutch chronic Q fever database. J Clin Microbiol.

[2] Maltezou HC, Raoult D (2002). Q fever in children. Lancet Infect Dis.

[3] Virk A, Mahmood M, Kalra M (2017). *Coxiella burnetii* multilevel disk space infection, epidural abscess, and vertebral osteomyelitis secondary to contiguous spread from infected abdominal aortic aneurysm or graft: report of 4 cases acquired in the US and review of the literature. Open Forum Infect Dis.

[4] Landais C, Fenollar F, Constantin A (2007). Q fever osteoarticular infection: four new cases and a review of the literature. Eur J Clin Microbiol Infect Dis.

[5] Eldin C, Mélenotte C, Mediannikov O (2017). From Q fever to Coxiella burnetii infection: a paradigm change. Clin Microbiol Rev.

[6] Israel Ministry of Health Weekly and periodic epidemiological reports for 2018.

[7] European Centre for Disease Prevention and Control (2019). Q Fever. ECDC. Annual Epidemiological Report for 2018.

